# Bacterial-Mediated Salinity Stress Tolerance in Maize (*Zea mays* L.): A Fortunate Way toward Sustainable Agriculture

**DOI:** 10.1021/acsomega.3c00723

**Published:** 2023-05-26

**Authors:** Baber Ali, Aqsa Hafeez, Muhammad Siddique Afridi, Muhammad Ammar Javed, Sumaira, Faiza Suleman, Mehwish Nadeem, Shehzad Ali, Mona S. Alwahibi, Mohamed S. Elshikh, Romina Alina Marc, Sezai Ercisli, Doaa Bahaa Eldin Darwish

**Affiliations:** † Department of Plant Sciences, Quaid-i-Azam University, Islamabad, Pakistan 45320; ‡ Department of Plant Pathology, Federal University of Lavras, Lavras, MG, Brazil 37200-900; § Institute of Industrial Biotechnology, Government College University Lahore, Lahore, Pakistan 54000; ∥ Department of Biotechnology, Quaid-i-Azam University, Islamabad, Pakistan 45320; ⊥ Department of Botany, Government College University Lahore, Lahore, Pakistan 54000; # Department of Botany, Government College University, Faisalabad 38000, Pakistan; ∇ Department of Environmental Sciences, Quaid-i-Azam University, Islamabad, Pakistan 45320; ○ Department of Botany and Microbiology, College of Science, King Saud University, Riyadh, Saudi Arabia 11451; ◆ Food Engineering Department, Faculty of Food Science and Technology, University of Agricultural Sciences and Veterinary Medicine of Cluj-Napoca, Cluj-Napoca, Romania 400372; ¶ Department of Horticulture, Agricultural Faculty, Ataturk Universitesi, Erzurum, Türkiye 25240; ⋈ Ata Teknokent, HGF Agro, TR-25240 Erzurum, Türkiye; ⧓ Botany Department, Faculty of Science, Mansoura University, Mansoura, Egypt 35511

## Abstract

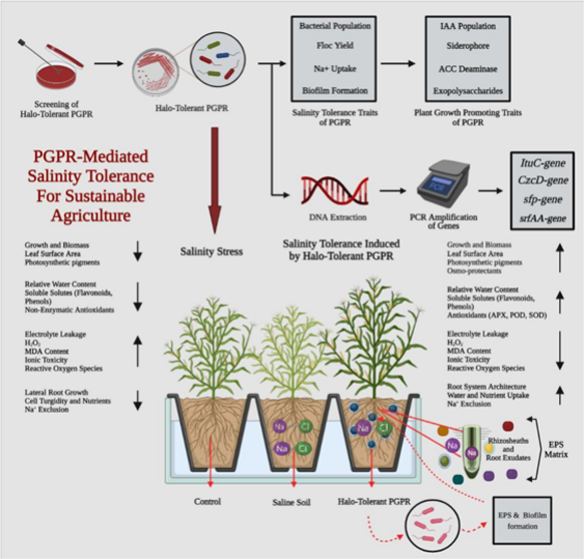

Sustainable agriculture is threatened by salinity stress because of the low yield quality and low crop production. Rhizobacteria that promote plant growth modify physiological and molecular pathways to support plant development and reduce abiotic stresses. The recent study aimed to assess the tolerance capacity and impacts of *Bacillus* sp. PM31 on the growth, physiological, and molecular responses of maize to salinity stress. In comparison to uninoculated plants, the inoculation of *Bacillus* sp. PM31 improved the agro-morphological traits [shoot length (6%), root length (22%), plant height (16%), fresh weight (39%), dry weight (29%), leaf area (11%)], chlorophyll [Chl *a* (17%), Chl *b* (37%), total chl (22%)], carotenoids (15%), proteins (40%), sugars (43%), relative water (11%), flavonoids (22%), phenols (23%), radical scavenging capacity (13%), and antioxidants. The *Bacillus* sp. PM31-inoculated plants showed a reduction in the oxidative stress indicators [electrolyte leakage (12%), H_2_O_2_ (9%), and MDA (32%)] as compared to uninoculated plants under salinity and increased the level of osmolytes [free amino acids (36%), glycine betaine (17%), proline (11%)]. The enhancement of plant growth under salinity was further validated by the molecular profiling of *Bacillus* sp. PM31. Moreover, these physiological and molecular mechanisms were accompanied by the upregulation of stress-related genes (APX and SOD). Our study found that *Bacillus* sp. PM31 has a crucial and substantial role in reducing salinity stress through physiological and molecular processes, which may be used as an alternative approach to boost crop production and yield.

## 1 Introduction

Abiotic stresses include drought,^1−3^ low or high temperatures,^4^ salinity,^5,6^ pesticides,^7^ heavy metals,^8−13^ minerals,^14−16^ and other environmental extremes. Abiotic stresses, especially salinity^17,18^ and drought,^19−22^ are some of the primary causes of crop loss worldwide.^23−25^ Soil salinity imposes major threats to food security^26^ and productivity of agricultural crops worldwide.^27^ High salinity stress increases ionic influxes, impaired antioxidant enzymes activities, photosynthesis,^17,28^ and free radicals, which lead to cellular malfunctions and consequently affect plant growth, biomass, and productivity.^29−32^ Soil salinity is a foremost concern in agriculture throughout the world and is affecting about 800 million hectares of cropland.^33^ It adversely affects different economically important crops such as wheat,^34^ soybean,^35^ and other crops, with the growth and productivity of maize being the most affected.^36^ High salinity promotes the utilization of an ethylene precursor ACC (1-aminocyclopropane-1-carboxylic acid deaminase), resulting in an increase in ethylene synthesis.^37^ Salinity also affects the Na^+^/K^+^ ratio and causes osmotic stress, which inhibits plant growth and development processes.^38,39^ The early seedling processes are affected by increased levels of salt in the rhizospheric region, which can ultimately deteriorate germination and vegetation parameters of several crops.^40^

Plants adapt to mechanisms of constitutive and induced resistance along with activation of different signaling pathways and induction of numerous genes to avoid or regulate salinity stress.^35,41,42^ Regulation of elevated ethylene synthesis can be useful to plants in reducing the detrimental effects of salt stress and improving agricultural yields even under saline environments.^43,44^ An outstanding salt tolerance ability is achieved by *Chenopodium album* by production of compatible solute and salt regulation.^45^ Similarly, neurotransmitters (dopamine and melatonin), cytosolic Ca^2+^, and reactive oxygen species (ROS) play a crucial role under salt stress in brassica to maintain ionic imbalances.^46^ Moreover, typical plant hormones, including abscisic acid (ABA), gibberellic acid (GA), cytokinins (CKs), salicylic acid (SA), jasmonates (JA), brassinosteroids (BRs), and triazoles (TR), are important growth regulators^47^ in plants and are all involved in the alleviation of salt stress directly or indirectly. For instance, the adverse effects of salinity were mitigated by the exogenous application of salicylic acid on maize plants.^48^ Plant growth regulators (vitamin E, vitamin C, BRs, JA) could be employed on rice crops to alleviate abiotic stresses.^49^

Nowadays, genetic engineering,^50,51^ genotyping,^52^ and plant growth-promoting bacteria (PGPR) are widely being used to mitigate abiotic stresses.^53,54^ Induction of PGPR is an ecofriendly method to increase salt tolerance and crop yield.^55^ Numerous bacterial species have been involved in the reduction of salt stress in plants.^56^ PGPR is a diversified group of microorganisms that have the ability to stimulate plant growth and development.^57^ PGPR colonizes the roots and improves seed germination and plant biomass production.^58^ Microorganisms alleviated abiotic stresses by modulating phytohormone levels.^59,60^ Plant growth, development, and nutrient acquisition^61−63^ were improved by these microbial phytohormones against various environmental stresses including both abiotic and biotic ones.^64−67^ The synthesis of phytohormones is one of PGPR’s other mechanisms for promoting plant growth and alleviating salt stress.^68^ Inoculation of PGPR in plants possessing ambient stresses enhances their proline level, which ultimately leads to improved antioxidant activity, thus increasing the production of plant biomass and improved toxicity.^69^ Inoculation of *Pseudomonas* sp. in salt-stressed *Lycopersicum esculentum* L. plants resulted in improved morphological traits, increased accumulation of osmolytes, phenol, and pigmented content, and increased antioxidant activities in salinity stress.^70^ The *Bacillus* genus has some species that have been reported widely as bacterization candidates because they regulate physiological characteristics, promote growth, and alleviate salinity stress in plants.^71^

**Figure 1:**
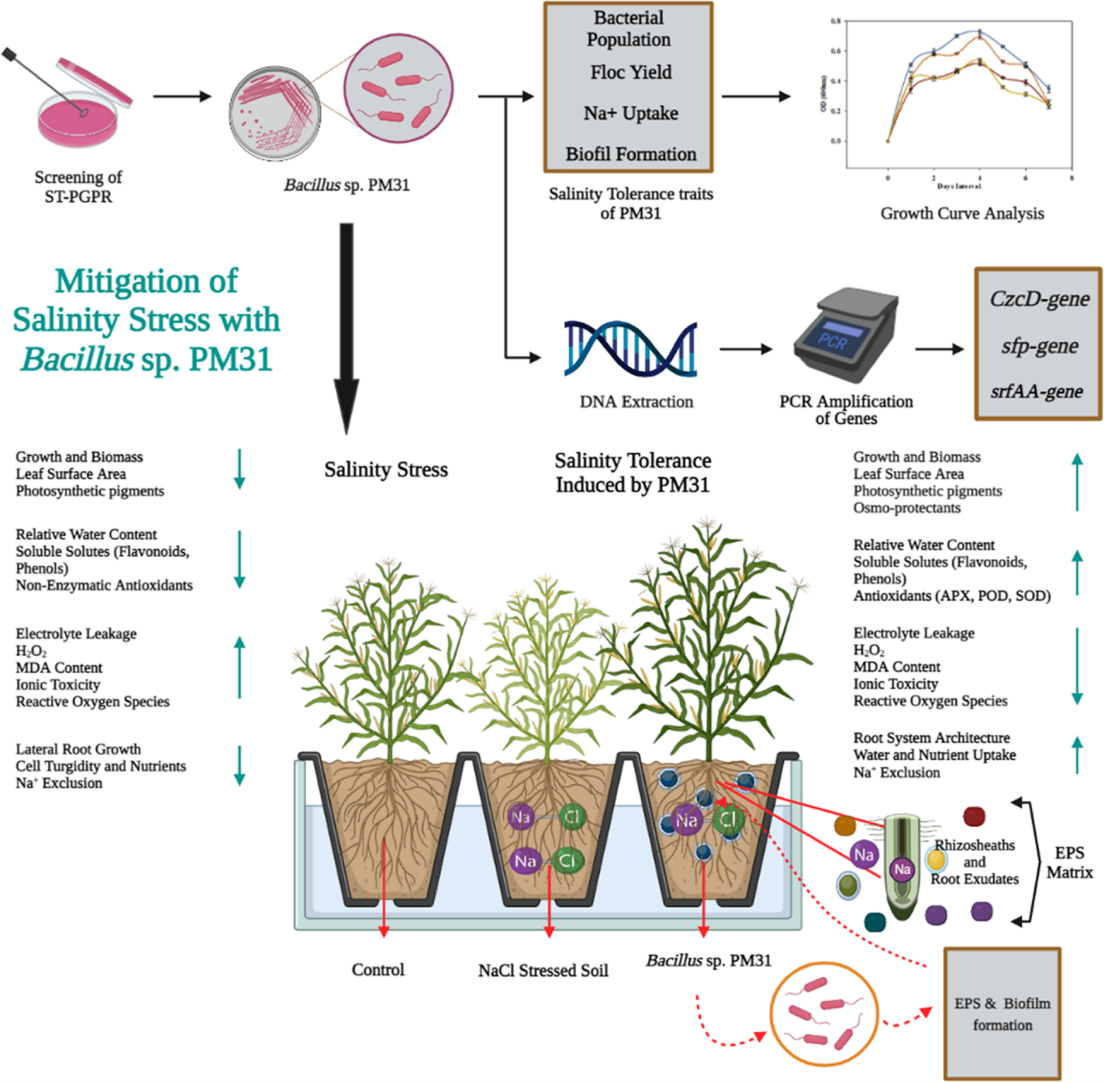
Brief summary of microbial-based salinity stress tolerance in maize.

The ACC deaminase-producing bacterial species provides plants with stress adaptability along with other mechanisms such as siderophores, indole-3-acetic acid (IAA) production, phosphorous and zinc solubilization, and ammonia and hydrogen cyanide (HCN) production, resulting in promotion of plant growth and development.^72^ The PGPB produces antioxidant enzymes and osmo-protector compounds, which are responsible for various physiological changes. Major changes include changes in the IAA level and the total protein, sugar, and ethylene contents, which result in systemically induced plant tolerance (IST).^73^*Bacillus subtilis* is a bacterium that has been used to relieve salt stress in *Zea mays* L. and promote plant development under salinity stress by producing the ACC deaminase enzyme.^74^ The ACC deaminase-producing bacteria inoculated in plants help in inducing abiotic stresses, salinity, and drought-tolerance ability.^75^ The use of maize is expanding more quickly in feed and wet milling sectors. Pakistan’s maize crop is largely cultivated in rain-fed regions.^76^ Maize has exposure to various abiotic constraints including salt stress, which restricts its growth, biomass, and productivity.^77^ Employing conventional breeding to improve crop resistance against salinity stress requires advanced technologies, continuous effort, and time.^78^ The isolation, screening, and selection of halo-tolerant PGPR comprise an ecofriendly^79^ and significant alternative to tackle salt stress problems in saline regimes.^79^ By keeping climate change and food security in mind, in this study, we investigated the tolerance potential, salinity tolerance traits, and impacts of *Bacillus* sp. PM31 on maize plants under salinity stress (Figure 1).

## 2 Materials and Methods

### 2.1 Procurement and Growth Curve Analysis of Bacteria

All bacterial strains (PM21, PM22, PM23, PM26, PM27, PM28, B29, B30, B31, B32, B33, and B38) were obtained from Plant-Microbe Interactions Lab, Quaid-i-Azam University, Islamabad, Pakistan. The strains were evaluated against salinity tolerance potential. The salt stress tolerance potential^80^ of *Bacillus* sp. PM31 was assessed. The TSB-broth supplemented with different concentrations of salinity stress (0, 1, 2, and 3 M NaCl) was prepared. The bacterium *Bacillus* sp. PM31 was inoculated in TSB-broth and incubated in a shaking incubator at 150 rpm and 32 ± 2 °C for 7 days to determine the tolerance potential. The optical density of each strain was measured at 600 nm daily for 7 days to evaluate the tolerance level of bacteria against salinity stress. The optical density (OD) of 0.5 was considered a salt-tolerant bacterial strain against salinity stress. The *Bacillus* sp. PM31 grew exponentially and showed resistance to salinity stress up to 3 M NaCl in both nutrient agar and broth media.

### 2.2 Salinity Tolerance Traits of *Bacillus* sp. PM31

The *Bacillus* sp. PM31 was exposed to different salt concentrations (0, 300, 600, and 900 mM NaCl) to evaluate salinity tolerance.^18^*Bacillus* sp. PM31 was cultivated in a TSB medium for 72 h at 30 °C to calculate bacterial flocculation.^81^ The *Bacillus* sp. PM31 was analyzed for its sodium uptake capacity under NaCl stress.^82^ Using the technique of crystal violet staining as suggested by O’Toole and Kolter,^83^ the biofilm-forming ability of *Bacillus* sp. PM31 was quantitatively assessed.

### 2.3 Quantification of PGP Traits of *Bacillus* sp. PM31

Indole-3-acetic acid was quantified through calorimetric methods.^84^ ACC deaminase and exopolysaccharides were quantified by following Ali et al.^41^ The siderophore production was performed using CAS (Chrome azurol S) agar media.^85^
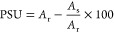
where *A*_s_ is the absorbance of the inoculated sample and *A*_r_ is the reference (uninoculated broth + CAS reagent + salt conc.).

### 2.4 Soil Analysis and Seed Inoculation

The soil for the experimental purpose was collected from the vicinity of Quaid-i-Azam University, Islamabad, Pakistan (33.7470°N, 73.1371°E). The soil’s physiochemical characteristics were evaluated accordingly. Maize seeds (variety SG-2002) were provided by National Agricultural Research Centre (NARC), Islamabad, Pakistan. Maize seeds were sterilized before sowing. The seeds were treated with a suspension solution of *Bacillus* sp. PM31. The seeds that had not been treated with the bacterial suspension were soaked in sterilized water for 2–4 h and regarded as controls.^18^

### 2.5 Experimental Layout

The maize seed variety (SG-2002) was surface-sterilized, and six seeds were sown in each pot filled with 200 g of sterilized soil. To prevent osmotic shock stress, the seedlings were subjected to salinity stress after five days of germination on daily bases with increments of 80, 120, and 180 mM NaCl until the desired salinity stress was achieved. Table S1 provides an overview of the experimental research design. Seeds were immersed in a bacterial suspension for up to 4 h, and the control was treated with distilled sterilized water. The pots containing sowing seeds were kept for 21 days in a growth chamber. The experimental design and growth conditions were followed as previously published.^18^

### 2.6 Agro-Morphological Traits of Maize

Plants were selected from each treatment including controls for examining the agro-morphological characteristics after 21 days. Plant leaves were kept in an 80 °C hot air oven for 24 h for drying, and their weights were recorded. A scale was used to determine the total leaf area.^86^

### 2.7 Photosynthetic Pigments

The photosynthetic pigments were quantified as follows.^87^
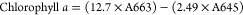

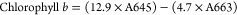

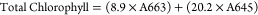

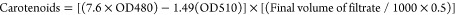


### 2.8 Leaf Water Content and Radical Scavenging Capacity of Maize

The relative water content (RWC) was measured by following Ali et al.^5^
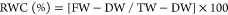
where FW is the fresh leaf weight, TW is the turgid leaf weight, and DW is the dry leaf weight.

The leaf extracts were used to assess the radical scavenging activities.^88^
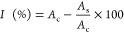
where *A*_c_ is the absorbance of the control and *A*_s_ is the absorbance of the sample.

### 2.9 Total Soluble Sugars (TSS), Proteins (P), and Antioxidant Enzymatic Assays

Total soluble sugars (TSS) were calculated using the Grad method.^89^ To generate the glucose standard curve, a stock solution of glucose was prepared in various concentrations (0, 20, 40, 60, and 100 mg), and optical density was measured at 625 nm. After absorbance, the regression model was used to generate the glucose standard curve. Bovine serum albumin (BSA), as described by Mendez et al.,^90^ was used as a reference to assess the protein content.

The antioxidants (POD, SOD, and APX) were evaluated in accordance with Hossain et al.^91^ and Afridi et al.^25^ The approach developed by El-Saadony et al.^92^ was utilized to calculate the ascorbic acid (AsA) content in the leaves of maize. The findings were calculated using a standard curve for ascorbic acid and are represented as mg/g FW.

### 2.10 Total Flavonoids (TFs), Phenolic Content (PC), Oxidative Burst, and Osmolytes

To determine the total flavonoid content, the method of Woisky and Salatino^93^ was used. The Folin-phenolic Ciocalteau reagent was used to spectrophotometrically quantify the phenols.^94^

By calculating the membrane stability index, electrolyte leakage was assessed.^95^
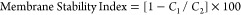
Endogenous H_2_O_2_ and malondialdehyde (MDA) concentrations were determined using the protocols of Kapoor et al.^96^ and Tulkova and Kabashnikova,^97^ respectively.
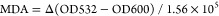
To detect free amino acids, Shafiq et al.^98^ established the ninhydrin technique. An earlier-described method was used to quantify the concentration of glycine betaine (GB).^99^ Proline content in shoots was measured using the technique proposed by Parveen and Siddiqui.^100^
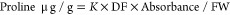


### 2.11 Polymerase Chain Reaction (PCR) Amplification of Genes

Iturin C (*ItuC*) gene detection was carried out using the universal primers (Table S2).^101^ In a 25 μL reaction mixture, the PCR reaction was conducted. Initial activation at 95 °C for 15 min, followed by 35 cycles of 95 °C for 1 min, 58 °C for 1 min, 72 °C for 1.5 min, and finally 72 °C for 7 min were the thermal cycling conditions. The *sfp* and *srfAA* genes were amplified using the primers (Table S2).^102,103^

### 2.12 Analysis of Gene Expression

The expression level of antioxidant genes (APX and SOD) was quantified by using quantitative real-time PCR (qRT-PCR) in the presence and absence of *Bacillus* sp. PM31 under salinity stress (0, 300, 600, and 900 mM NaCl). The Qiagen RNeasy Plant Mini kit (cat. nos. 74903, Hilden, Germany) was used to isolate total RNA from the leaves of maize plants and to remove the contaminated DNA. First-strand cDNA was synthesized using the Qiagen Reverse Transcription kit. The cDNA was then diluted to a final volume of 500 μL with ddH_2_O, and real-time assays were performed with SYBR Green I Master (Roche, Basel, Switzerland) according to the manufacturer’s recommendations. Reaction efficiency values were calculated by running each primer set on serial dilutions of a cDNA mixture comprising stressed and control plants.

Real-time quantitative PCR (RT-qPCR) was carried out using the Qiagen RNeasy Plant Mini kit (cat. nos. 74903, Hilden, Germany) with gene-specific primers designed for SOD (F: 5′-ACATTTGCTACCTCTCCCTCACCT-3′ R: 3′-TCGGGTAAGACATCGTCGGTATGT-5′), and APX (F: 5′-AAACCCAAGCTCAGAGAGCCTCAT-3′ R: 3′-TACTTCACGGTGCTTCTTGGTGGA-5′). Reaction mixture recipes are described in Table S3. The PCR amplification conditions were optimized in accordance with Hu et al.^104^ The level of gene expression was assessed using the housekeeping gene Actin and the 2^–ΔΔCt^ technique.^105^

### 2.13 Statistical Analysis

The standard errors and mean values used in the experiment were computed. The Statistix 8.1 program was used to perform analysis of variance on all of the obtained data, and the LSD test was utilized to generate a pairwise comparison between all of the mean values (*p* = 0.05). For computed data, principal component analysis (PCA) and Pearson correlation analysis were performed using the R program.

## 3 Results

**Figure 2:**
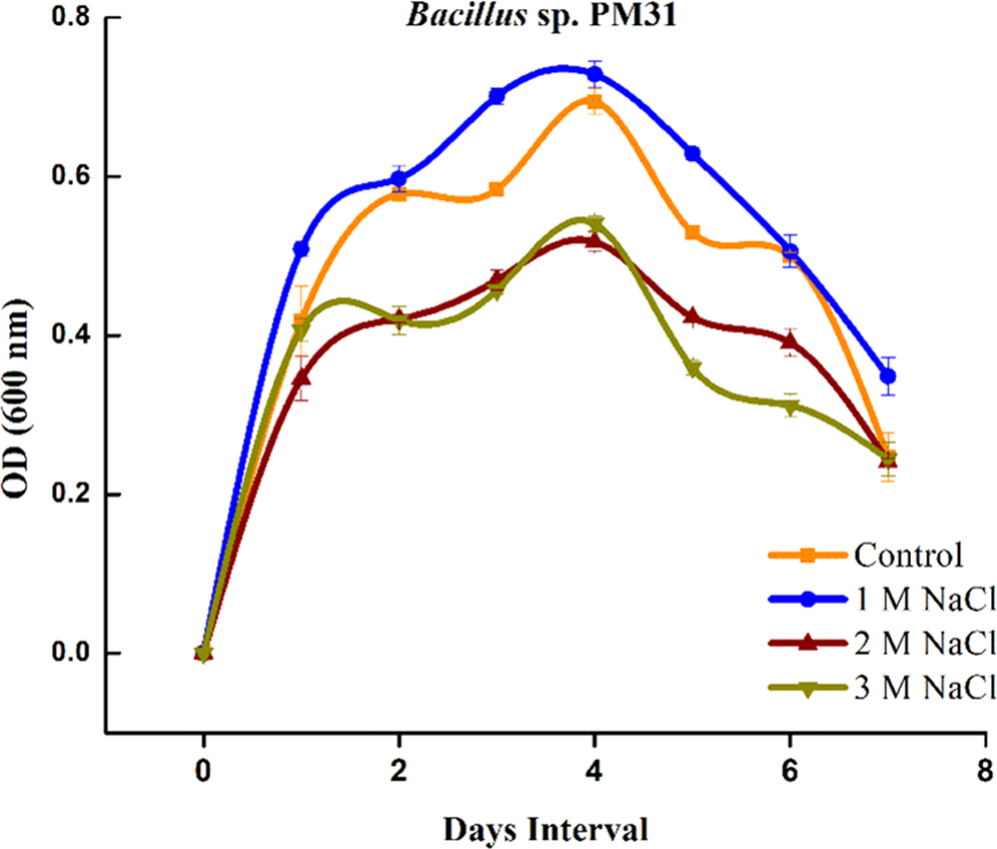
Growth curve analysis of *Bacillus* sp. PM31 under salinity stress.

**Figure 3:**
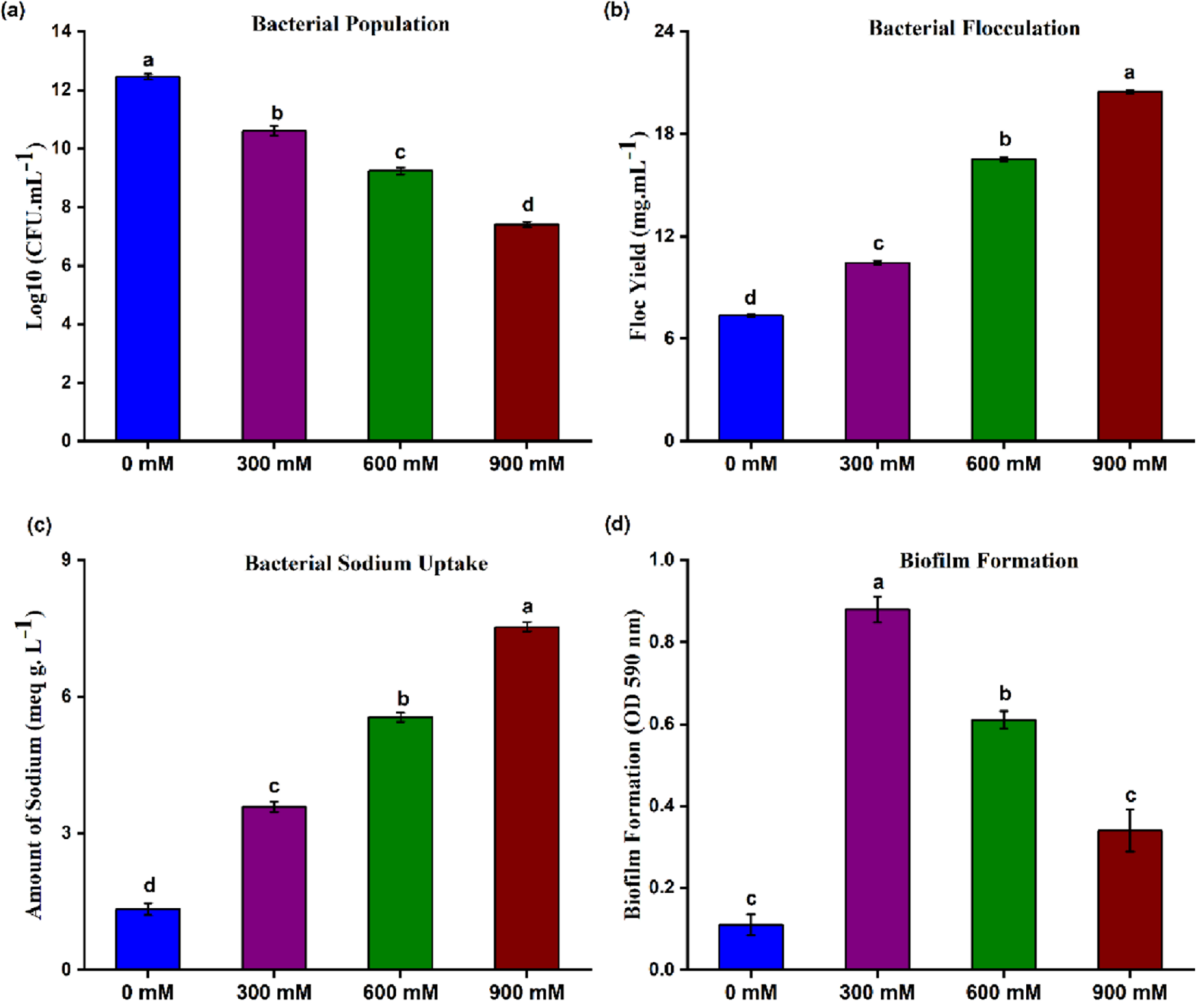
Effects of NaCl on salinity tolerance traits of *Bacillus* sp. PM31. (a) Bacterial population. (b) Flocculation yield. (c) Bacterial Na^+^ uptake. (d) Biofilm formation. Bars sharing different letter(s) for each parameter are significantly different from each other according to the least significant difference (LSD) test (*p* ≤ 0.05). All of the data represented are the average of three replications (*n* = 3). Error bars represent the standard errors (SEs) of three replicates.

**Figure 4:**
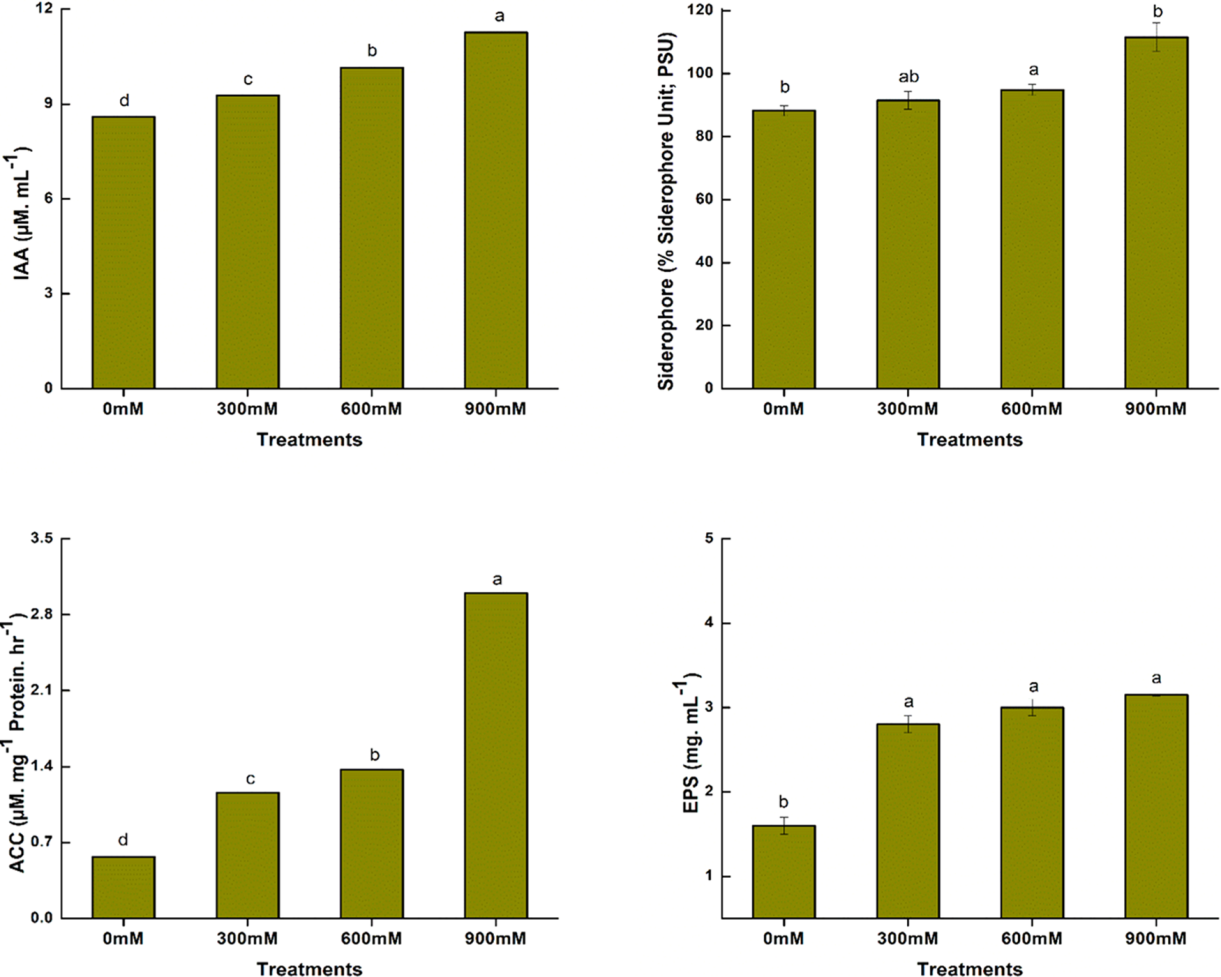
Quantitative estimation of different PGP traits of *Bacillus* sp. PM31 under salinity stress.

It was investigated that *Bacillus* sp. PM31 can endure salinity stress and showed healthy growth in salt-amended LB-media. *Bacillus* sp. PM31 can resist NaCl concentration of up to 3 M (Figure 2). The CFU (colony-forming unit) values were used to determine the bacterial growth. As the salinity increased, the bacterial population continued to decline in number. Hence, salinity stress and bacterial growth showed an inverse relation as compared to the control (Figure 3a). It means that the growth of the bacterial population declined with the increasing salinity stress. However, a direct relation was observed in terms of biomass when the NaCl concentration led to a remarkable increase in the floc yield. At 900 mM NaCl, PM31 showed the significantly highest floc yield (Figure 3b). Similarly, sodium absorption by *Bacillus* sp. PM31 was significantly higher at 900 mM NaCl with a value of 7.53 meq/L (Figure 3c). The maximum biofilm production for *Bacillus* sp. PM31 was observed at 300 mM NaCl (Figure 3d). By the production of phytohormones, siderophores, and exopolysaccharides and decreasing the ethylene production via ACC deaminase, these PGPR alleviate the salinity stress. The quantification of PGP traits of *Bacillus* sp. PM31 increased with progressive salinity stress (Figure 4). The results showed that PGP traits were directly proportional to NaCl concentrations.

**Figure 5:**
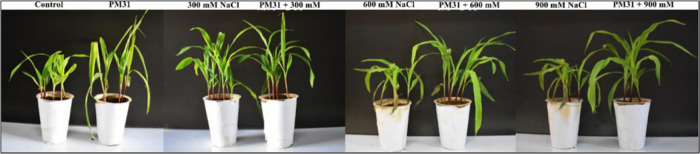
Effect of *Bacillus* sp. PM31 on plant growth promotion of *Zea mays* L. under salinity stress.

The physiochemical traits of soil were previously published.^5^ The maize plants were carefully harvested after 21 days to check the impacts of *Bacillus* sp. PM31 on growth and biomass production (Figure 5, Table 1). Under conditions of salinity stress, a significant decline was recorded in agro-morphological traits of maize plants as compared to control plant samples. When maize plants were given a concentration of 900 mM salinity stress, substantial reductions in agro-morphological characteristics were found. The maize plants inoculated with *Bacillus* sp. PM31 showed much better growth and yield than uninoculated plants (Table 1). A significant increment in shoot length (14–30%), root length (8–22%), plant height (16–27%), fresh weight (28–39%), dry weight (23–29%), and leaf surface area (8–19%) was observed. PM31-inoculated maize plants, on the other hand, exhibited more promising growth and biomass output than controlled ones (Table 1).

**Table 1:** Agro-Morphological Traits of *Zea mays* L. in the Presence and Absence of *Bacillus* sp. PM31 under Salinity Stress^a^

NaCl (mM)	*Bacillus* sp. PM31	shoot Length (cm)	root Length (cm)	plant height (cm)	fresh weight (g)	dry weight (g)	leaf area (cm^2^)
0 mM	–PM31	25.4 ± 0.38 c	15.63 ± 0.42 ab	41.03 ± 0.09 c	1.49 ± 0.04 bcd	0.27 ± 0.008 bcd	13.57 ± 0.70 b
	+PM31	36 ± 0.70 a	17.06 ± 0.36 a	53.06 ± 0.58 a	2.15 ± 0.15 a	0.36 ± 0.01 a	16.75 ± 0.002 a
300 mM	–PM31	21.6 ± 0.52 d	13.13 ± 0.47 bc	34.73 ± 0.85 d	1.38 ± 0.08 cde	0.24 ± 0.006 cd	11.43 ± 0.002 e
	+PM31	31 ± 0.26 b	16.44 ± 0.24 a	47.44 ± 0.24 b	1.91 ± 0.02 ab	0.31 ± 0.009 ab	12.45 ± 0.006 f
600 mM	–PM31	20.34 ± 0.34 de	10 ± 0.70 de	30.34 ± 1.00 e	1.14 ± 0.03 de	0.21 ± 0.004 cd	10.30 ± 0.002 d
	+PM31	25 ± 0.26 c	11.41 ± 0.34 cd	36.31 ± 0.08 d	1.76 ± 0.03 abc	0.28 ± 0.01 bc	11.82 ± 0.04 g
900 mM	–PM31	18 ± 0.26 e	6.5 ± 0.56 f	24.5 ± 0.41 f	1.00 ± 0.05 e	0.15 ± 0.002 e	8.59 ± 0.004 h
	+PM31	21 ± 0.26 d	8.3 ± 0.34 ef	29.3 ± 0.09 e	1.63 ± 0.04 bc	0.21 ± 0.01 d	9.61 ± 0.04 g

a Growth was measured at 21 days after seed sowing under different salt concentration regimes. The treatments exhibiting dissimilar letters within rows represent significance (*p* ≤ 0.05).

The maize (*Z. mays* L.) plants showed a significant increase in proteins, while a significant decrease in pigmented content was observed under salinity stress as compared to control plants (Table 2). The highest decrement in pigments, carotenoids, and TSS and significant increment in antioxidant activity and total protein content were observed at 900 mM NaCl as compared to control plants (0 mM NaCl). By the application of *Bacillus* sp. PM31, maize plants significantly enhanced the chlorophyll content (*chl a*: 6–20%; *chl b*: 23–37%; *total chl*: 14–22%), carotenoids (8–18%), and total soluble sugars (23–43%), while increased DPPH activity (13–28%) and total protein content (38–41%) were observed as compared with uninoculated plants under salinity stress (Table 3). Moreover, salt-tolerant (ST)-PGPR-treated plants revealed higher values for pigmented content, carotenoids, DPPH activity, and protein and sugar contents as compared to uninoculated plants (Table 2).

**Table 2:** Pigmented Contents (Chl) and Carotenoids, Antioxidant Activity (DPPH, %), Total Soluble Sugars, and Total Protein Content in *Zea mays* L. Leaves in the Presence and Absence of *Bacillus* sp. PM31 under Salinity Stress^a^

NaCl (mM)	*Bacillus* sp. PM31	Chl *a* (mg/g FW)	Chl *b* (mg/g FW)	total Chl (mg/g FW)	carotenoids (mg/g FW)	DPPH (IC_50_) %	soluble sugars (mg/g FW)	proteins (mg/g FW)
0 mM	–PM31	15.6 ± 0.02 ab	4.31 ± 0.21 cd	19.91 ± 0.20 b	5.43 ± 0.05 abc	33.20 ± 0.95 e	64.59 ± 1.53 d	0.28 ± 0.01 e
	+PM31	16.56 ± 0.02 a	6.50 ± 0.11 a	23.06 ± 0.11 a	5.88 ± 0.26 a	45.86 ± 0.93 cd	84.15 ± 0.88 a	0.45 ± 0.004 c
300 mM	–PM31	12.37 ± 0.002 de	4.04 ± 0.12 cde	16.41 ± 0.12 c	4.65 ± 0.002 cde	40.45 ± 0.95 de	53.91 ± 1.03 e	0.34 ± 0.01 d
	+PM31	14.63 ± 0.02 bc	5.52 ± 0.10 b	20.15 ± 0.22 d	5.69 ± 0.03 ab	49.44 ± 0.85 bc	80.36 ± 0.52 ab	0.58 ± 0.005 b
600 mM	–PM31	10.36 ± 0.26 fg	3.47 ± 0.10 e	13.83 ± 0.10 c	4.18 ± 0.04 ef	46.07 ± 0.79 cd	47.24 ± 1.02 f	0.37 ± 0.01 d
	+PM31	12.98 ± 0.55 cd	4.50 ± 0.09 c	17.49 ± 0.60 e	5.00 ± 0.15 bcd	54.62 ± 1.06 b	77.25 ± 0.66 bc	0.63 ± 0.005 ab
900 mM	–PM31	8.97 ± 0.002 g	2.26 ± 0.12 f	11.23 ± 0.12 d	3.77 ± 0.002 f	55.48 ± 0.96 b	41.14 ± 0.64 f	0.40 ± 0.01 cd
	+PM31	10.79 ± 0.40 ef	3.61 ± 0.04 de	14.40 ± 0.41 i	4.43 ± 0.13 def	64.00 ± 1.95 a	71.61 ± 1.33 c	0.67 ± 0.004 a

a Chl *a*, chlorophyll *a;* Chl *b*, chlorophyll *b*; Total Chl, total chlorophyll; and carotenoids, DPPH–2,2-diphenyl-1-picrylhydrazyl. The treatments exhibiting dissimilar letters within rows represent significance (*p* ≤ 0.05).

**Table 3:** Relative Water Content, Flavonoids, and Phenolic Content in the Presence and Absence of *Bacillus* sp. PM31 under Salinity Stress^a^

NaCl (mM)	*Bacillus* sp. PM31	RWC (%)	TFC (mg QE/g FW)	TPC (mg GAE/g FW)
0 mM	–PM31	59.56 ± 0.37 b	109.96 ± 0.97 b	10.17 ± 0.40 cd
	+PM31	65.57 ± 0.35 a	122.07 ± 1.31 a	13.22 ± 0.28 a
300 mM	–PM31	55.56 ± 0.35 c	96.16 ± 0.79 d	9.65 ± 0.23 cde
	+PM31	60.54 ± 0.21 b	104.68 ± 0.56 bc	12.51 ± 0.12 a
600 mM	–PM31	48.52 ± 0.16 e	86.85 ± 0.56 e	8.67 ± 0.33 de
	+PM31	55.48 ± 0.35 c	99.42 ± 2.29 cd	12.02 ± 0.01 ab
900 mM	–PM31	46.53 ± 0.23 e	74.13 ± 0.81 f	8.09 ± 0.43 e
	+PM31	52.49 ± 0.36 d	94.52 ± 1.19 de	10.54 ± 0.12 bc

a RWC, relative water content; TFC, total flavonoid content; TPC, total phenolic content; QE, quercetin; GAE, gallic acid; FW, fresh weight. The treatments exhibiting dissimilar letters within rows represent significance (*p* ≤ 0.05).

**Figure 6:**
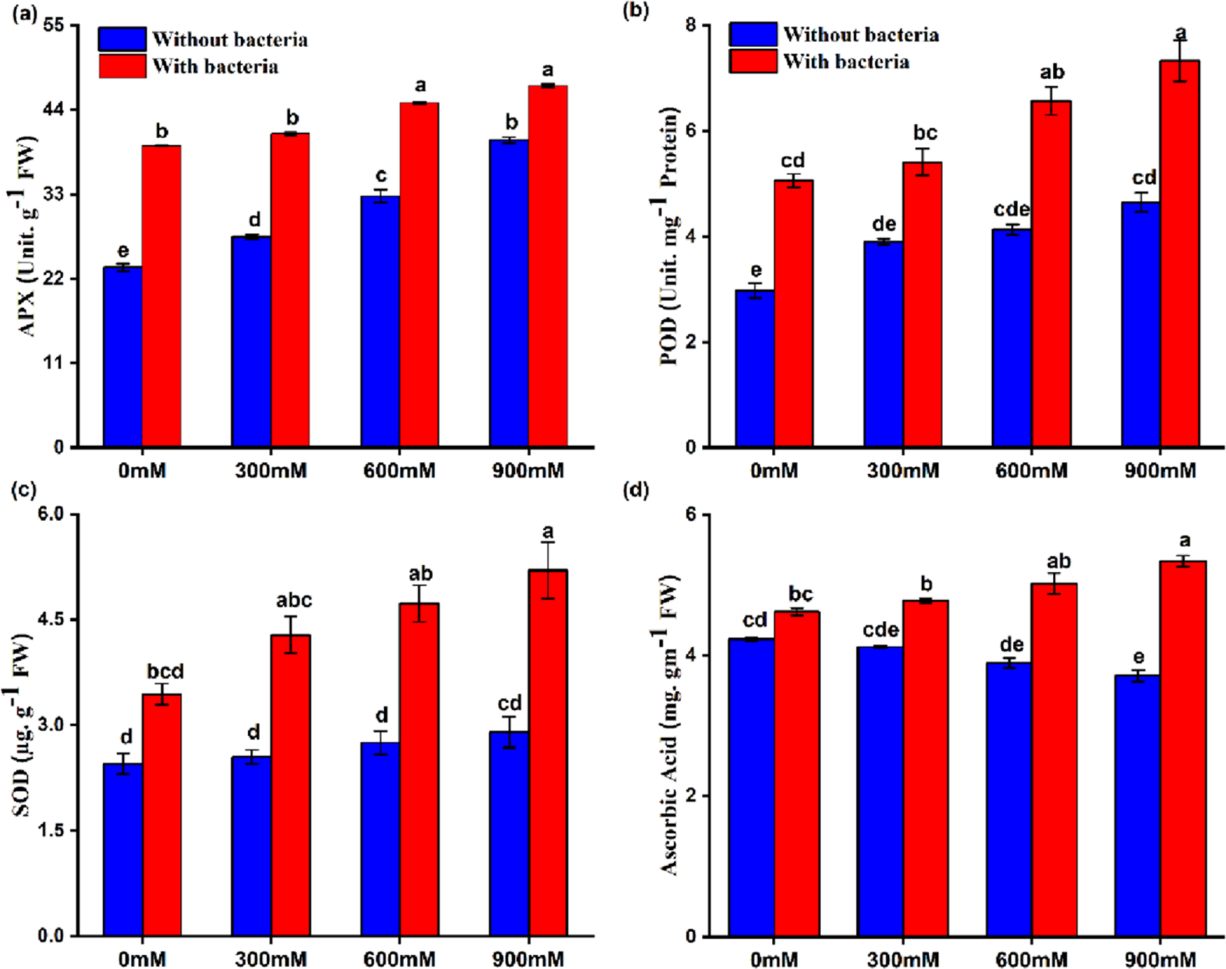
Effects of *Bacillus* sp. PM31 on the levels of enzymatic and nonenzymatic antioxidants; (a) APX, (b) POD, (c) SOD, and (d) ascorbic acid. Bars sharing different letter(s) for each parameter are significantly different from each other according to the least significant difference (LSD) test (*p* ≤ 0.05). All of the data represented are the average of three replications (*n* = 3). Error bars represent the standard errors (SEs) of three replicates.

The maize plants were analyzed for RWC under salinity (Table 3). A higher water content (11%) was expressed in PM31-inoculated maize plants as compared to uninoculated plants (Table 3). The effects of *Bacillus* sp. PM31 on antioxidants, flavonoids, and phenolic levels of maize were studied (Figure 6, Table 3). Enzymatic antioxidants were significantly increased, while ascorbic acid, flavonoids, and phenols decreased under salt stress as compared to the control. A significant increment in enzymatic and decrement in nonenzymatic antioxidants, flavonoid, and phenols were recorded (Table 3). However, the inoculation of *Bacillus* sp. PM31 in *Z. mays* L. plants resulted in significant increases in the levels of APX, POD, SOD, and ascorbic acid (Figure 6), while the total flavonoid (8–22%) and phenolic content (23–28%) showed significant alleviation with respect to the uninoculated ones (Table 3).

Salinity asserted a remarkable impact on osmolytes and oxidative burst (Table 4). As compared to controls, the oxidative burst including ELL, H_2_O_2_, and MDA content and osmolyte levels increased significantly under salinity stress (Table 4). However, with the application of *Bacillus* sp. PM31, inoculated plants showed a decrease in ELL (12–16%), H_2_O_2_ (9–21%), and MDA (32–37%) contents and significant increases in the levels of FAA (34–48%), GB (17–30%), and proline (11–18%) as compared with noninoculated ones (Table 4). ELL, H_2_O_2_, and MDA remarkably decreased, while osmolytes increased (Table 4).

**Table 4:** Levels of Different Oxidative Stress Markers and Osmoprotectants in the Presence and Absence of *Bacillus* sp. PM31 under Salinity Stress^a^

NaCl (mM)	*Bacillus* sp. PM31	ELL (%)	H_2_O_2_ (μmol/g FW)	MDA (nmol/g FW)	amino acid (mg/g DW)	GB (μg/g DW)	proline (μmol/g FW)
0 mM	–PM31	39.23 ± 0.33 d	22.54 ± 0.52 de	8.21 ± 0.37 d	10.96 ± 0.31 f	5.55 ± 0.11 e	64.51 ± 0.12 g
	+PM31	33.12 ± 0.21 e	18.39 ± 0.26 f	5.55 ± 0.13 e	21.27 ± 0.54 c	7.94 ± 0.01 abc	76.84 ± 0.29 e
300 mM	–PM31	44.66 ± 0.40 bc	25.98 ± 0.70 bc	9.78 ± 0.17 bc	13.56 ± 0.23 e	6.53 ± 0.12 de	69.48 ± 0.10 f
	+PM31	38.94 ± 0.50 d	20.54 ± 0.30 ef	6.48 ± 0.15 e	24.57 ± 0.28 b	8.17 ± 0.43 abc	85.07 ± 0.41 c
600 mM	–PM31	47.79 ± 0.72 ab	29.52 ± 0.51 a	10.7 ± 0.13 b	16.39 ± 0.19 d	7.27 ± 0.08 cd	75.05 ± 0.15 e
	+PM31	41.56 ± 0.54 cd	25.20 ± 0.45 cd	6.73 ± 0.11 e	26.07 ± 0.41 ab	8.77 ± 0.27 ab	88.77 ± 0.25 b
900 mM	–PM31	50.12 ± 1.34 a	31.03 ± 0.33 a	12.63 ± 0.15 a	18.47 ± 0.39 d	7.61 ± 0.11 bcd	82.49 ± 0.12 d
	+PM31	43.9 ± 0.72 bc	28.39 ± 0.34 ab	8.65 ± 0.27 cd	28.14 ± 0.54 a	9.16 ± 0.16 a	93.09 ± 0.64 a

a ELL, electrolyte leakage; H_2_O_2_, hydrogen peroxide; MDA, malondialdehyde; GB, glycine betaine. The treatments exhibiting dissimilar letters within rows represent significance (*p* ≤ 0.05).

**Figure 7:**
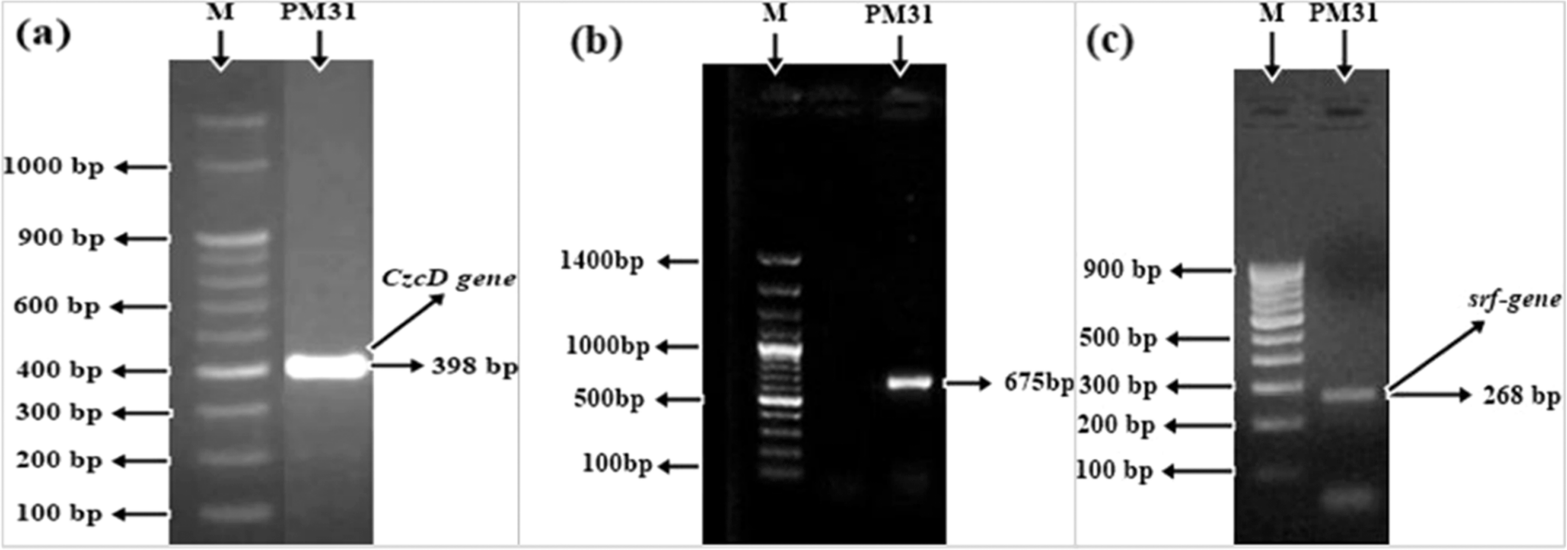
Amplification of abiotic stress-related genes in *Bacillus* sp. PM31. (a) Metal-resistant related *CzcD* gene; (b) surfactant-producing *sfp* gene; (c) *srfAA* gene.

**Figure 8:**
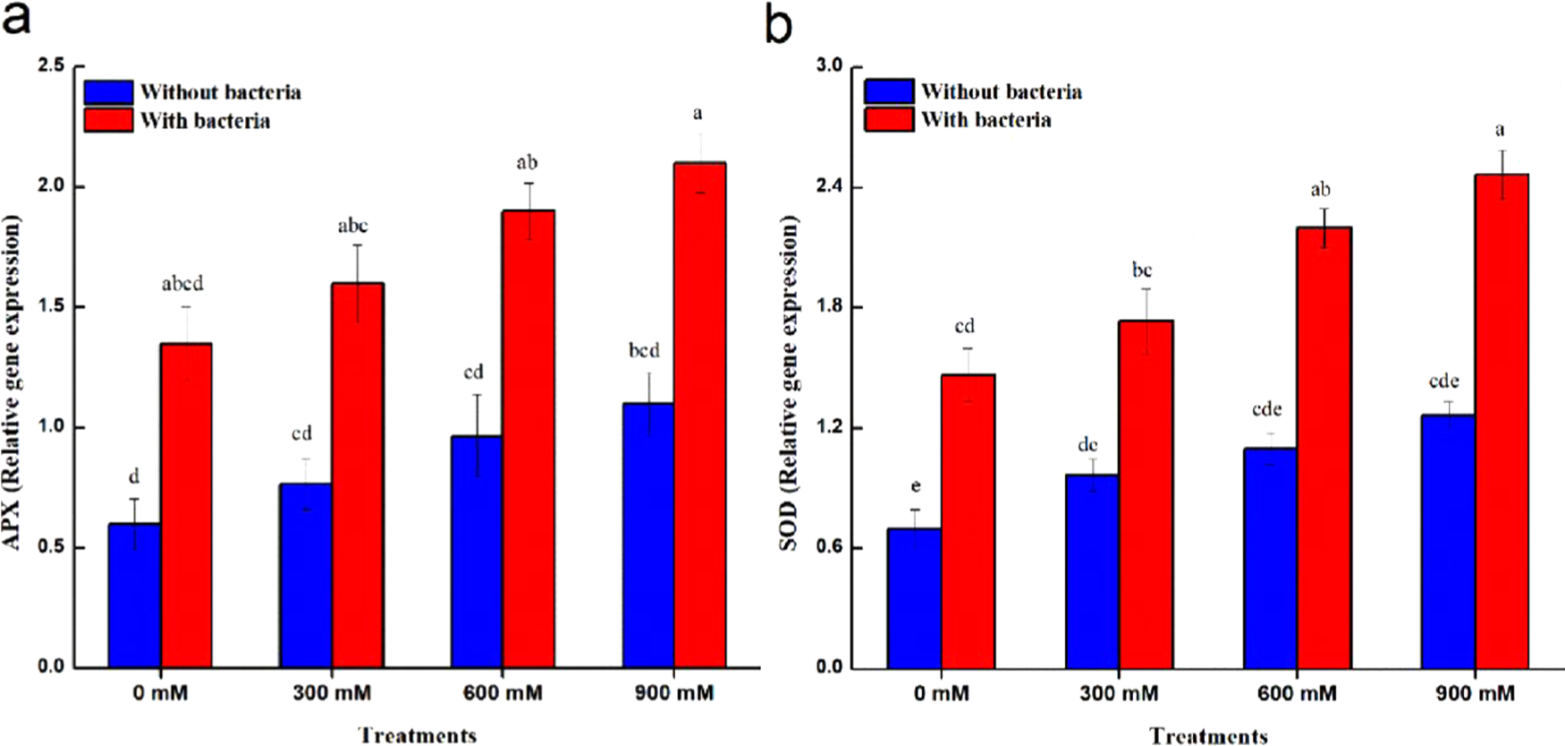
Expression levels of antioxidant genes of maize in the absence and presence of *Bacillus* sp. PM31 under salinity stress. (a) Ascorbate peroxidase (APX). (b) Superoxide dismutase (SOD). Bars sharing different letter(s) for each parameter are significantly different from each other according to the least significant difference (LSD) test (*p* ≤ 0.05). All of the data represented are the average of three replications (*n* = 3). Error bars represent the standard errors (SEs) of three replicates.

Abiotic stress-related genes in *Bacillus* sp. PM31 were amplified. PCR amplification of the halo-tolerant bacteria was performed for *CzcD* (398 bp), *sfp* (675 bp), and *srfAA* (268 bp) genes using a set of primers (Table S2), which resulted in sharp bands (Figure 7). Under control conditions, *Bacillus* sp. PM31 inoculation increased the expression of antioxidant genes (APX and SOD) as compared to uninoculated controls (Figure 8). Moreover, *Bacillus* sp. PM31-inoculated salinity-stressed maize showed greater antioxidant expression and salt-tolerant genes with respect to uninoculated plants.

**Figure 9:**
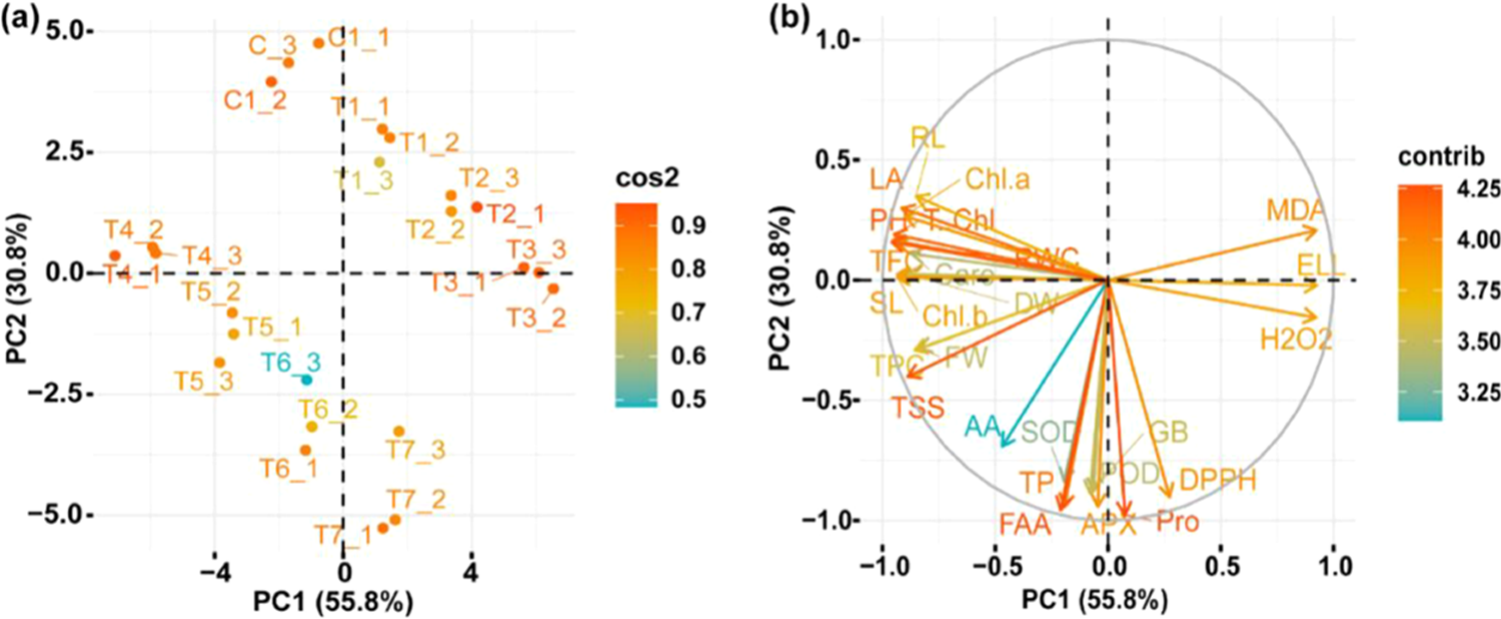
Categorization of *Bacillus* sp. PM31 based on its effects on maize growth-promoting characteristics under salinity stress. (a) Cluster analysis. (b) PCA biplot analysis.

**Figure 10:**
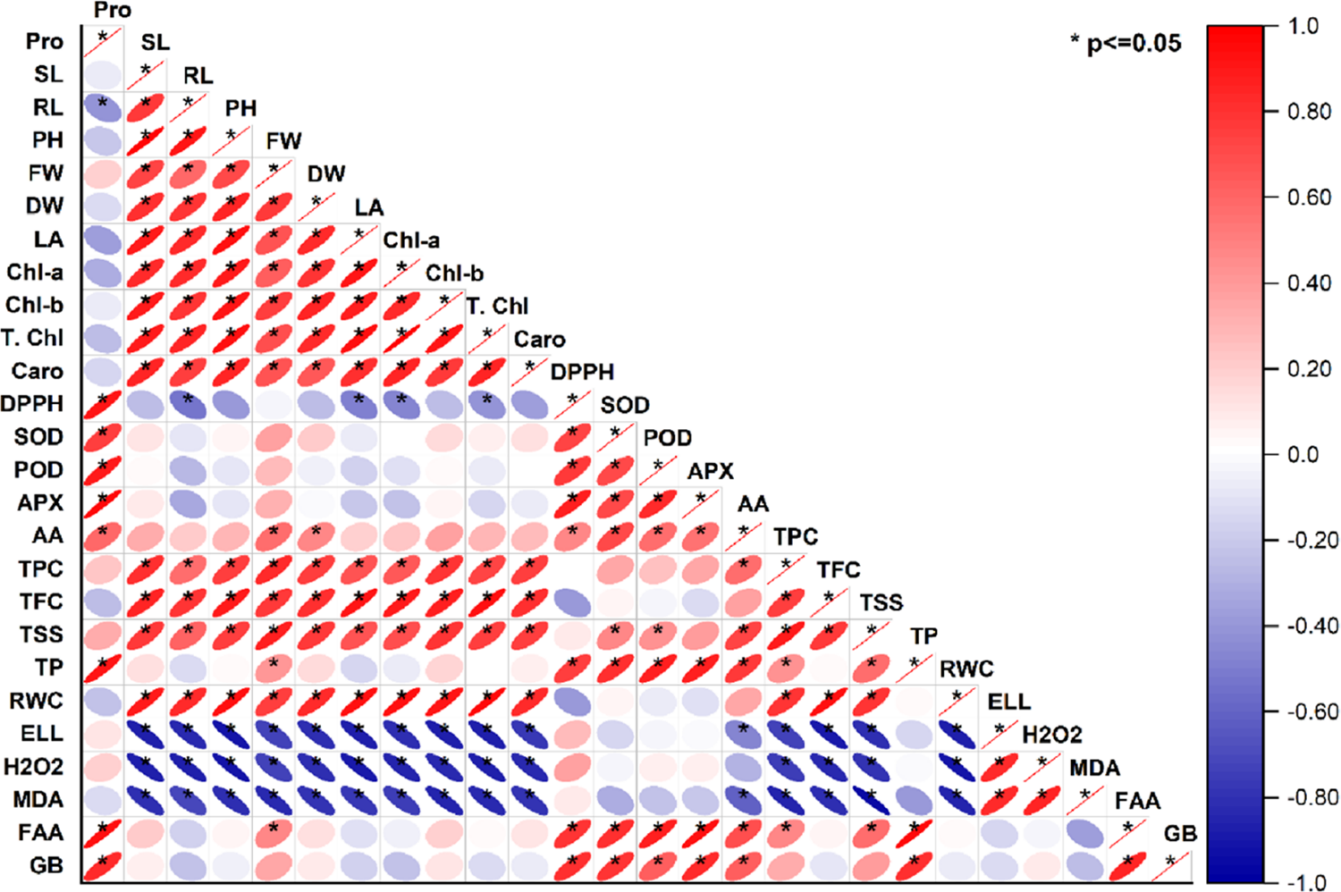
Pearson’s correlation between antioxidants and biochemical traits with plant biomass parameters under various salt stresses: Pro (proline), SL (shoot length), RL (root length), PH (plant height), FW (fresh weight), DW (dry weight), LA (leaf area), Chl *a* (chlorophyll *a*), Chl *b* (chlorophyll *b*), T. chl (total chlorophyll), Caro (carotenoids), DPPH (radical scavenging capacity), SOD (superoxide dismutase), POD (peroxidase), APX (ascorbate peroxidase), AA (ascorbic acid), TPC (total phenolic content), TFC (total flavonoid content), TSS (total soluble sugars), TP (total protein), RWC (relative water content), ELL (electrolyte leakage), H_2_O_2_ (hydrogen peroxide), MDA (malondialdehyde), FAA (free amino acid), and GB (glycine betaine).

The principal component analysis showed the impacts of PM31 on the growth and physiology of maize plants under salinity stress. The biplot indicated 81.5% variance (PC_1_ = 59.0%; PC_2_ = 22.5%) (Figure 9). Pearson’s correlation also illustrated the effects of *Bacillus* sp. PM31 under stress conditions in maize plants (Figure 10). The plant growth showed high correlation with antioxidants and osmoprotectants and negative correlation with oxidative stress markers.

## 4 Discussion

Soil is a complex medium^106^ comprising a huge number of a variety of microorganisms such as fungi, bacteria, and archaea.^107^ Some microbes have plant growth-promoting abilities along with their abiotic stress (salinity stress) tolerance capabilities. Nowadays, owing to the increasing population, food security is a critical issue.^108^ The present investigation was focused on assessing the salinity tolerance traits and potential of *Bacillus* sp. PM31 to mitigate salinity stress in maize. *Bacillus* spp. is a well-known plant growth promoter and has great resistance potential against salt stress to serve as a PGPR. *Bacillus* sp. is well-known for its growth-promoting qualities and elimination of salinity stress.^109^

In a recent investigation, we reported *Bacillus* sp. PM31 to be showing salinity tolerance potential up to 3 M salt concentration in LB-broth. In this study, *Bacillus* sp. PM31 had shown a low bacterial population rate at 900 mM NaCl-modified media, which is similar to that in a previous report of Qurashi and Sabri.^110^ Salinity stress can have a significant impact on the bacterial population in the soil. A high salt concentration can reduce the diversity and abundance of bacterial populations in the soil as many bacteria are not able to tolerate high levels of salt.^111^ This can result in an altered composition of the bacterial community, with the presence of salt-tolerant bacteria increasing and the presence of salt-sensitive bacteria decreasing. This can lead to changes in the overall function of the soil microbial community, which can impact nutrient cycling, organic matter decomposition, and other important ecosystem processes.^112^ In addition, the production of EPS by some bacteria can help maintain the soil structure and the water-holding capacity, making the soil less susceptible to erosion and drought.^113^ Therefore, salt stress can have a major impact on the functioning of soil ecosystems and the provision of ecosystem services.

Bacterial sodium uptake refers to the process by which bacteria take up sodium ions from their surrounding environment. Sodium ions play an important role in maintaining the osmotic balance of bacteria and in supporting various cellular processes, including the maintenance of the membrane potential and the transport of nutrients and waste products.^114^ Some bacteria are capable of adapting to high salt concentrations by actively taking up sodium ions and maintaining a low internal salt concentration.^115^ This allows them to survive in environments with high salt levels. Other bacteria are unable to regulate their internal salt concentration and are sensitive to high levels of salt, which can lead to cell death.^116,117^ Bacterial sodium uptake is an important process in soil ecosystems, as high salt levels can occur in many soils, particularly in arid and semiarid regions. Understanding how bacteria take up sodium ions and how they are affected by salt stress is crucial for understanding the functioning of soil ecosystems and the provision of ecosystem services.

Formation of biofilms might be considered a protection strategy under abiotic stresses.^118^ The biofilm production is regulated by nutrients present in the medium (Figure 3). The nutrients in the media are in less concentration and that is why biofilm formation is reduced.^119^ It was reported that *Bacillus* spp. has promising effects on growth and yield and proved to be tolerant against abiotic stresses.^120^ Plant growth-promoting bacteria (PGPB) have been shown to promote biofilm formation under salt stress conditions as they can help plants cope with salt stress by improving their tolerance.^121^ Plant growth-promoting bacteria (PGPB) are known to produce various plant growth-promoting compounds, such as phytohormones and enzymes, which can help plants overcome the adverse effects of salt stress.^122^ Additionally, PGPB can form biofilms on plant roots, which can help reduce the uptake of harmful salt ions, protect roots from osmotic stress, and provide plants with essential nutrients. Biofilm formation can also increase the ability of PGPB to fix nitrogen and solubilize phosphorus, both of which are critical for plant growth and survival. Therefore, by promoting biofilm formation, PGPB can play an important role in mitigating the negative impacts of salt stress on plant growth and health.^123,124^

Salinity reduces the crop yield, particularly in dry and semiarid regions.^125^ Furthermore, it creates metabolic abnormalities and reduces the meristematic activity and plant cell elongation, which leads to a higher respiration rate and inhibition of growth.^126^ In our study, inoculation of *Bacillus* sp. PM31 to maize plants significantly enhanced plant growth, biomass, and leaf surface area as compared to noninoculated plants. Soil treated with PGPR increased the growth parameters of maize plants through the excretion of various phytohormones in the plant’s rhizosphere coupled with strong endogenous control.^127^ PGPR helps in the elongation of root cells and their proliferation by IAA production.^128^ According to our results, *Bacillus* sp. PM31 could be described as PGPR because it assisted in the improvement of maize root growth and development. This PGPR helped maintain normal plant growth during salt stress by increasing the water and nutrient uptake in maize roots. The phytohormone synthesis increased the root shape and physiology, resulting in better water and food absorption.^129^ Thus, a link may be established between salt-tolerant PGPR and enhanced agronomical parameters and maize plant biomass, inferring that these traits not only mitigate salinity stress but also enhance growth attributes. Plant–microbe interaction is a useful combination,^130^ providing a more efficient and ecofriendly strategy for the reclamation of salt-affected soils to enhance revegetation efficiency and crop productivity.

Under salinity stress, chlorophyll content is an important indicator of plant development and photosynthetic efficiency.^41,131^ Salinity stress reduces photosynthetic activity because of structural and functional damage to the electron carriers and photosystems.^42^ An equilibrium between the osmotic quality of the cytosol and vacuole is maintained by the accumulation of soluble sugars.^132^

According to our findings, as salt stress levels increased, the pigmented content decreased; however, on concurrent treatment with *Bacillus* sp. PM31, these metrics increased significantly under salinity stress. This increment might be because of reduced levels of oxidative stress markers and a better antioxidant system, relative water, flavonoids, and phenolic content that assist in the improvement of biosynthesis of the photosynthetic pigments resulted in an increase of biomass, growth parameters, and yield. Salt accumulation reduces the synthesis of pigments, leading to reduced enzyme activity.^133^ Salinity stress reduces chlorophylls, leaf water potential, and photosynthesis while increasing salt absorption.^134^ Increased production of phytohormones, EPS, and ACC deaminase by PGPR aids in the increased water and nutrient uptake and their transport to vegetative parts for participation in enhanced pigment production.^128^

PGPR treatment altered the cell wall structure and physio-biochemical changes that lead to protein and enzyme production, which are associated with pigment stability.^135^ PGPR application not only increased the carotenoid content but also has a dual role in inducing carotenoid protection and preventing carotenoid oxidation by ROS.^136^ Furthermore, the functional activity of PSII may be used to assess a plant’s photosynthetic capacity. The application with *Bacillus* sp. PM31 under salt stress increased the effectiveness of photosynthesis and gaseous exchange of the leaf. Previous reports have also shown the same results in *Arabidopsis* plants treated with *Pseudomonas knackmussii* MLR6 under salinity stress.^137^

The recent findings demonstrated that *Bacillus* sp. PM31 produced more compatible solutes (soluble sugars) in a medium enriched with high NaCl (900 mM). Several investigations have shown that bacteria could accumulate a considerable amount of compatible solutes inside their cells, which helps them survive under severe osmotic stress.^138^ These compatible solutes help the plant maintain the water level under saline conditions. Ge and Zhang^139^ showed that inoculation of cucumber seedlings with *Rhodopseudomonas palustris* G5 increased the soluble sugar content under salt stress (3% NaCl).

The generation of ROS in plants cell leads to oxidative stress, which impairs the functionalities of chlorophyll, DNA, proteins, and membranes and alters the antioxidative defense systems of plants.^140^ Salinity stress caused a significant reduction in the growth and productivity of maize plants in a recent study. Both salinity and the plant-beneficial rhizospheric microbe (PBRM) have an impact on the antioxidant capabilities of maize.^141^ Results demonstrated that inoculation with *Bacillus* sp. PM31 augmented maize plants’ DPPH activity. This is in line with the findings of Kilam et al.,^142^ who found that inoculating *Stevia rebaudiana* with *Piriformospora indica* and PGPR together improved the plant’s ability to scavenge radicals. Moreover, a 24% increase in the scavenging capacity of *Enterobacter*-inoculated tomato plants has been shown in inoculated plants.^143^

Leaf RWC is a critical measure in determining the water status indexes of plants and the extent to which plants can tolerate environmental stress. Salinity stress frequently impacts a plant’s water status and reduces the hydraulic conductivity of the roots, resulting in reduced root-to-shoot water flow.^134^ According to this study, reduced root-to-shoot water flow exerted detrimental impacts on maize plants owing to salinity and decreased RWC. On the other hand, treatment with *Bacillus* sp. PM31 eliminated the Na^+^-ion content, which helped in the increased absorption of water and RWC under salinity conditions. It was observed by Mayak et al.^144^ that PGPR significantly increases root development by increasing RWC under salinity stress.^145^

Salinity stress produces ROS such as superoxide and hydrogen peroxide, and these ROS cause damage to lipids and proteins.^146^ This illustrated that *Bacillus* sp. PM31 can assist the plant in combating ROS-induced damage caused by salt stress. These findings were in line with prior research showing a correlation between increased antioxidant enzyme activity and increased salt tolerance.^147^ Increasing the activity of antioxidant enzymes mitigated the oxidative damage induced by abiotic stress like salinity and drought.^148^

Salinity stress causes H_2_O_2_-mediated membrane peroxidation that disturbs the stability of cell membranes.^149^ Similarly, this study also shows that under soil salinity electrolyte leakage, ROS levels (MDA and H_2_O_2_ content) were increased. However, inoculation with *Bacillus* sp. PM31 decreased MDA and H_2_O_2_ in maize. The production of chlorophyll molecules is affected by oxidative stress markers linked with increased Na^+^ absorption and decreased Mg accumulation.^150^ Moreover, H_2_O_2_ translocation harms the DNA, proteins, and lipids.^151^ Oxidative stress damages the plasma membrane and disturbs the chlorophyll levels via excessive H_2_O_2_ production.^152^ Likewise, PGPR-inoculated maize reduces the ELL, MDA, and H_2_O_2_ under salinity.^87^

MDA is a widely employed physiological index for the determination of cell membrane stability and permeability, influenced by the increased extent of oxidative damage.^141^ In our study, application of *Bacillus* sp. PM31 caused a significant reduction in MDA with respect to the uninoculated control under salinity stress. The beneficial microorganisms reduce the negative effects of oxidative stress to protect the cell membranes.^153,154^

Due to various environmental constraints, plants build up various organic osmolytes such as proline, polyamines, and quaternary ammonium complexes.^155^ From the results, halo-tolerant *Bacillus* sp. PM31-inoculated plants were found to improve compatible solute levels under salinity stress. These outcomes were in line with those of Wang et al.^156^ These findings indicated that osmoprotectants provide an effective mechanism to save plants from salt stress. Plants produce osmolytes, which help keep the membrane structure healthy by reducing the formation of ROS and preserving the membrane.^157^

In the current investigation, surfactants producing *srfAA* and *sfp* genes for *Bacillus* sp. PM31 were amplified. Surfactants assisted tomato plants in maintaining their ionic homeostasis, particularly lowering the Na^+^ absorption, and therefore enhanced tomato growth under salinity stress.^158^ The *CzcD* gene was also amplified, which encodes metal resistant in *Bacillus* sp. PM31.^159^

Furthermore, inoculation of maize plants with *Bacillus* sp. PM31 massively improved the gene expression linked with salinity tolerance and antioxidant enzymes. The regulation of two genes APX and SOD caused an alleviation of salinity stress in chickpeas.^160^ Salinity stress was also mitigated in rice seedlings when the levels of antioxidants were increased by the inoculation of PGPB.^161^

## 5 Conclusions

The current study found a significant surge in the ability to resist salinity stress after inoculating maize plants with halo-tolerant PGPR (*Bacillus* sp. PM31). The halo-tolerant PGPR treatment markedly alleviates salinity stress. This bacterium possesses plant growth-promoting characteristics, and when applied as a liquid formulation, it stimulates maize growth as well as an increase in biomass under saline stress conditions. This rhizospheric bacterium was critical in alleviating saline stress in maize plants by improving the antioxidant defense system, lowering oxidative stress indicators, and by accumulation of compatible solutes. Findings of the current study suggested that halo-tolerant PGPR has the potential to be used as an alternative and environmentally acceptable medium, facilitating maize production and mitigating saline stress. The present study in a pot experiment with the inoculation of PGPR at various salinity stress conditions gives a baseline for analysis of potential PGPR under salinity stress. Altogether, *Bacillus* sp. PM31 improves plant growth along with increasing salinity tolerance, and it can be used as a biofertilizer. The PGPR verify the efficacy and full potential of salinity stress mitigation and support for large-scale implementation in sustainable agriculture. However, further study *in vitro* on natural salinity conditions under field circumstances is necessary.

## Supporting Information Available

Experimental design, primers used for gene amplifications, and PCR recipe for real-time analysis (PDF)
