# Supplementary material for: Bacterial-Mediated Salinity Stress Tolerance in Maize (Zea mays L.): A Fortunate Way toward Sustainable Agriculture
Source: ACS Omega. 2023 May 26;8(23):20471–87. doi: 10.1021/acsomega.3c00723 (PMC10275368; doi:10.1021/acsomega.3c00723)
Supplement: Supplementary file 1 — ao3c00723_si_001.pdf [file ao3c00723_si_001.pdf]

Bacterial-Mediated Salinity Stress Tolerance in Maize (*Zea mays* L.): A Fortunate Way towards Sustainable Agriculture

Baber Ali *[1]†, Aqsa Hafeez [1]†, Muhammad Siddique Afridi [2], Muhammad Ammar Javed [3], Sumaira [4], Faiza Suleman [5], Mehwish Nadeem [6], Shehzad Ali [7], Mona S. Alwahibi [8], Mohamed S. Elshikh [8], Romina Alina Marc [9], Sezai Ercisli [10, 11*], Doaa Bahaa Eldin Darwish [12]

1. Quaid-i-Azam University, Department of Plant Sciences, Islamabad, PK 45320; aqsahafeez@bs.qau.edu.pk (A.H)
2. Federal University of Lavras, Department of Plant Pathology, Lavras, MG, BR 37200-900; msiddiqueafridi@gmail.com (M.S.A)
3. Government College University Lahore, Institute of Industrial Biotechnology, Lahore, PK 54000; ammarjaved94@gmail.com (M.A.J)
4. Quaid-i-Azam University, Department of Biotechnology, Islamabad, PK 45320; sumaira.khan1890@gmail.com (S)
5. Government College University Lahore, Department of Botany, Lahore, PK 54000; faizasuleman9803@gmail.com (F.S)
6. University of Agriculture Faisalabad, Department of Botany, Faisalabad, Punjab, PK 38000; mehwishnadeem1234@gmail.com
7. Quaid-i-Azam University, Department of Environmental Sciences, Islamabad, PK 45320; alishahzad7899@gmail.com
8. King Saud University, Department of Botany and Microbiology, College of Science, Riyadh, SA 11451; wamona015@gmail.com (M.S.A); melshikh@ksu.edu.sa (M.S.E)
9. University of Agricultural Sciences and Veterinary Medicine of Cluj-Napoca, Food Engineering Department, Faculty of Food Science and Technology, Cluj-Napoca, RO 400372; romina.vlaic@usamvcluj.ro (R.A.M)
10. Ataturk Universitesi, Department of Horticulture, Agricultural Faculty, Erzurum, TR 25240, sercisli@atauni.edu.tr (S.E)
11. HGF Agro, Ata Teknokent, TR-25240 Erzurum, Turkey
12. Mansoura University, Botany Department, Faculty of Science, Mansoura, EG 35511; d_darwish@mans.edu.eg

(†)These authors share first authorship.

***Correspondence:** baberali@bs.qau.edu.pk (B.A); sercisli@atauni.edu.tr (S.E)

Table S1. Experimental design for pot studies

Treatments	Description
T0	Control
T1	300 mM NaCl
T2	600 mM NaCl
T3	900 mM NaCl
T4	*Bacillus* sp. PM31
T5	PM31 + 300 mM
T6	PM31 + 600 mM
T7	PM31 + 900 mM

Table S2. Primers Used for Gene Amplifications

Genes	Primers	Primer Sequence (5′-3′)	PCR Product Size Expected/Detected (bp)
ItuC	*ITUC-F1*	5′-CCCCCTCGG TCAAGTGAATA-3′	506
	ITUC-R1	5′-TTGGTTAAG CCCTGATGCTC-3′	
sfp	*sfp F*	5′ATGAAGATTTACGGAATTTA-3′	675
	sfp R	5′-TTATAAAAGCTCTTCGTACG-3′	
srfAA	*srfAA F*	F-5′-TCGGGACAGGAAGACATCAT-3′	268
	srfAA R	R-5′-CCACTCAAACGGATAATCCTGA-3′	

Table S3. PCR recipe for real-time analysis.

Component	Volume (per Reaction)
Sterile ddH_2O	3 µL
Primer (forward + reverse) 10mM	2 µL
SYBR Green I Master (Roche) 2X	10 µL
cDNA Template	5 µL
**Total Volume**	**20 µL**
